# Web-Based Educational Intervention to Improve Knowledge of Systematic Reviews Among Health Science Professionals: Randomized Controlled Trial

**DOI:** 10.2196/37000

**Published:** 2022-08-25

**Authors:** Marina Krnic Martinic, Marta Čivljak, Ana Marušić, Damir Sapunar, Tina Poklepović Peričić, Ivan Buljan, Ružica Tokalić, Snježana Mališa, Marijana Neuberg, Kata Ivanišević, Diana Aranza, Nataša Skitarelić, Sanja Zoranić, Štefica Mikšić, Dalibor Čavić, Livia Puljak

**Affiliations:** 1 University Hospital Split Split Croatia; 2 Catholic University of Croatia Zagreb Croatia; 3 Department of Research in Biomedicine and Health University of Split School of Medicine Split Croatia; 4 University of Split School of Medicine Split Croatia; 5 University North Varazdin Croatia; 6 Faculty of Health Studies University of Rijeka Rijeka Croatia; 7 University Department of Health Studies University of Split Split Croatia; 8 Department of Health Studies University of Zadar Zadar Croatia; 9 Department of Nursing University of Dubrovnik Dubrovnik Croatia; 10 Faculty of Dental Medicine and Health Osijek The Josip Juraj Strossmayer University of Osijek Osijek Croatia

**Keywords:** educational intervention, systematic review, health science professionals, knowledge, randomized controlled trial

## Abstract

**Background:**

Lack of knowledge of systematic reviews (SRs) could prevent individual health care professionals from using SRs as a source of information in their clinical practice or discourage them from participating in such research.

**Objective:**

In this randomized controlled trial, we evaluated the effect of a short web-based educational intervention on short-term knowledge of SRs.

**Methods:**

Eligible participants were 871 Master’s students of university health sciences studies in Croatia; 589 (67.6%) students who agreed to participate in the trial were randomized using a computer program into 2 groups. Intervention group A (294/589, 49.9%) received a short web-based educational intervention about SR methodology, and intervention group B (295/589, 50.1%) was presented with the PRISMA (Preferred Reporting Items for Systematic Reviews and Meta-Analyses) checklist. The participants’ knowledge of SRs was assessed before and after the intervention. The participants could not be blinded because of the nature of the intervention. The primary outcome was the difference in the percentage of correct answers about SR methodology per participant between the groups after the intervention, expressed as relative risk and 95% CI.

**Results:**

Results from 162 and 165 participants in the educational intervention and PRISMA checklist groups, respectively, were available for analysis. Most of them (educational intervention group: 130/162, 80.2%; PRISMA checklist group: 131/165, 79.4%) were employed as health care professionals in addition to being health sciences students. After the intervention, the educational intervention group had 23% (relative risk percentage) more correct answers in the postintervention questionnaire than the PRISMA checklist group (relative risk=1.23, 95% CI 1.17-1.29).

**Conclusions:**

A short web-based educational intervention about SRs is an effective tool for short-term improvement of knowledge of SRs among health care studies students, most of whom were also employed as health care professionals. Further studies are needed to explore the long-term effects of the tested education.

**Trial Registration:**

OSF Registries 10.17605/OSF.IO/RYMVC; https://osf.io/rymvc

## Introduction

### Background

Evidence-based medicine (EBM), which is interchangeably also called evidence-based practice (EBP) or evidence-based health care (EBHC) [1], is credited with a major impact on health care [2]. Systematic reviews (SRs) are considered the gold standard evidence that helps in making decisions about health within the concept of EBM [3].

However, multiple studies have shown a low level of knowledge of EBM among health care professionals. Low awareness of EBM was reported by Novak et al [4] among physicians in Croatia, and limited knowledge but a positive attitude toward EBM was reported by Ulvenes et al [5] among Norwegian physicians. A study conducted by Munroe et al [6] showed that only 3% of nurses evaluated their knowledge of EBP as very good.

Knowledge of SRs is considered important for health sciences and medical students as well because it is important that clinicians know how to find and appraise evidence [7]. Knowledge of SRs in trainees can help not only in developing useful skills in critical appraisal but also in addressing important clinical questions and serve as a strong basis to design new, original research studies that will fill the gaps and answer relevant and unsolved clinical questions [8].

The importance of medical students’ exposure to EBM was shown by Vrdoljak et al [9], who reported that knowledge and attitudes of mentors toward EBM in general practice can be influenced by using medical students as academic detailers. It has been shown that better knowledge and more positive attitudes toward EBM among medical students are associated with the exposure to the vertical subject on research in biomedicine and activities of The Cochrane Collaboration [10]. Glass et al [11] reported that summarized research evidence delivered in a poster format can increase student nurses’ access to the evidence base. This intervention has increased their knowledge to guide their clinical practice. Thus, knowledge of EBM is a variable that can be influenced. A lack of knowledge of SRs and EBM could prevent individual health care professionals from using SRs as a source of information in their clinical practice or discourage them from participating in such research. Several studies have shown the effectiveness of educational programs on changing the beliefs on and attitudes toward EBM of health care professionals and their readiness to use evidence from EBM sources such as the Cochrane Library or SRs to solve clinical problems [12-15].

Web-based educational interventions are low-cost, easy to implement, easily refined and stored for later use, and easily accessible by health care professionals. Educational interventions conducted via the internet related to various topics in medicine have been shown to be effective [16,17]. Several studies have also proved the effectiveness of web-based educational interventions among health care professionals on knowledge of EBP [12-14].

A 2017 Campbell SR on the effectiveness of e-learning in improving knowledge of EBHC showed that, compared with no learning, pure e-learning improved knowledge of and skills regarding EBHC but not attitudes and behaviors [18]. Varnell et al [12] showed that an accelerated 8-week training program influenced a statistically significant positive change in beliefs on and attitudes toward EBP. A controlled trial examining the effect of an educational intervention on knowledge of EBM among physicians in Israel [14] reported a significant improvement in the level of knowledge of and attitudes toward EBM but not a significant impact on clinical practice [14].

### Objectives

We were not able to find studies evaluating the effectiveness of educational interventions dedicated to learning about SRs and SR methodology. In this randomized controlled trial (RCT), we evaluated the effect of a short web-based educational intervention about SRs on short-term knowledge of SR among students of health sciences studies in Croatia.

## Methods

### Ethics Approval

The study protocol was approved by the Ethics Committee of the Catholic University of Croatia on March 1, 2021 (Klasa: 641-03/21-01/03; Urbroj: 498-03-02-06-02/1-21-02). Subsequently, the ethics committees of all participating institutions also approved the study protocol. The participants provided written informed consent to take part in the study.

### Guidelines for Reporting

The manuscript was reported in line with the CONSORT (Consolidated Standards of Reporting Trials) checklist [19]. The CONSORT checklist for this manuscript is available in Multimedia Appendix 1. The educational intervention was reported in line with the Guideline for Reporting Evidence-based Practice Educational Interventions and Teaching (GREET) checklist [20].

### Trial Registration

The study protocol was prospectively registered (ie, before enrolling the first participants) on the Open Science Framework website [21]. There were no differences between the protocol and the conducted trial.

### Study Design

We conducted an RCT with 2 parallel groups and 1:1 participant allocation.

### Participants

#### Inclusion Criteria

The participants were students of Master’s university health sciences studies in Croatia. The study programs available at the participating universities were Nursing, Radiological Technology, Clinical Nutrition, Physiotherapy, and other programs. Full-time and part-time students were eligible to take part in the study. Many of these students were already employed in health care; students were eligible for participation regardless of their employment status.

#### Institutions

There were 8 eligible institutions in Croatia for this study, and we invited all of them. The following 7 institutions accepted the invitation to participate: Catholic University of Croatia; University Department of Health Studies Split; University Department of Health Studies Zadar; University of Dubrovnik, Nursing Studies; University North, Faculty of Dental Medicine and Health; University of Osijek, Faculty of Health Studies; and University of Rijeka. One institution declined the invitation to participate in the study (University of Zagreb School of Medicine).

#### Contacting the Students

Students from eligible institutions were contacted via email by coauthors (MC, MN, KI, DA, NS, SZ, and SM) employed in these institutions and invited to participate in the study on brief web-based education about SRs of the literature. Students who agreed to participate were randomized by simple randomization using the Randomizer website. After randomization, they were sent an email invitation to access the web-based platform on which materials for participants from the educational intervention and PRISMA (Preferred Reporting Items for Systematic Reviews and Meta-Analyses) checklist groups were available.

The text of the email provided information about the study and provisions related to the anonymity of the participants according to the General Data Protection Regulation, and students were invited to click on the link to further participate in the study. For this study, 2 separate interfaces for the participants were created on the SurveyMonkey platform (Momentive Inc). One interface was created for participants in the educational intervention group and the other for participants enrolled in the PRISMA checklist group. Each group accessed their interface using a separate link.

The link in the email took the participants to their respective SurveyMonkey web-based interface. The text of the email to the participants is presented in Multimedia Appendix 2. In the SurveyMonkey interface, the participants were initially asked to confirm that they voluntarily took part in the research and that they were providing informed consent to participate in the study by entering the next page.

### Intervention Group A

In the web-based interface, intervention group A received a newly developed intervention created by the authors of this study with expertise in medical and health sciences education and research methodology. The educational intervention was written in the Croatian language. It consisted of 11 short educational texts on the methodology of producing SRs. A module describing the forest and funnel plots contained figures of those 2 graphs. The content of the educational intervention was an abbreviated version of the information contained in Cochrane’s educational materials for web-based learning about SRs of the literature (Cochrane Interactive Learning). The complete content of the educational intervention, translated into English, is presented in Multimedia Appendix 3.

The learning objectives of the educational intervention anticipated that the participants would be able to define EBM, recognize different levels of evidence, define an SR, ask a clinical question, define the steps for preparing and registering an SR protocol, describe literature search and screening, explain the risk-of-bias assessment, and describe the process of data analysis and interpretation in SRs. In addition to theory, there was a practical learning objective: the participants were expected to be able to differentiate between an abstract of an SR and of a narrative literature review.

The first version of the educational intervention was iteratively revised within the team. Before conducting this trial, the web-based interface with the educational intervention was evaluated in a qualitative study among health care workers via semistructured interviews (Krnic Martinic et al, unpublished data, November 2021). The results of the users’ feedback obtained in the qualitative study were used to revise the educational intervention.

The intervention was delivered as an asynchronous web-based education that did not include any components of live education or interaction.

The participants were able to go back and forth through the web-based interface with the educational modules and respective questions without a time limit.

### Intervention Group B

Intervention group B was presented with the PRISMA checklist [22] for reporting on SRs (Multimedia Appendix 4) in their web-based interface, and the participants were asked to read it. It was presented to the participants in 11 separate sections to be as similar as possible to the number and form of the educational texts in intervention group A.

### Pre- and Postintervention Questionnaires

Both groups completed a preintervention questionnaire containing questions about demographic characteristics and their knowledge of SRs before the presentation of the intervention (educational intervention or PRISMA checklist group; Multimedia Appendix 5). We were unable to find questionnaires on this topic and purpose in the literature. Thus, we designed the pre- and postintervention questionnaires specifically for this study. The pre- and postintervention questionnaires were not validated. Questions evaluating knowledge of SRs were based on the questions used in our previous studies on knowledge of SRs [23] and the definitions of SRs [24].

At the end of the educational intervention or PRISMA checklist presentation, the participants were asked to answer the postintervention questionnaire (Multimedia Appendix 5). The questionnaire contained the same questions on knowledge of SRs as in the preintervention questionnaire as well as questions about whether they agreed with the proposed characteristics of the definition of SRs. Finally, they were presented with 4 abstracts of published articles and asked to assess whether they were abstracts of SRs.

As part of the postintervention questionnaire, the participants were asked to express the level of their agreement on whether an SR should have 6 characteristics proposed earlier by Krnic Martinic et al [24]. They were asked to express their agreement with a number on a Likert scale ranging from 1 to 5 that best suited their opinion, where 1 meant *I do not agree at all* and 5 meant *I completely agree* (Multimedia Appendix 5).

After those questions, the participants were presented with 4 abstracts of published scientific articles, of which 2 (50%) were abstracts of SRs [25,26] and the other 2 (50%) were abstracts of narrative reviews of the literature [27,28]. They were chosen based on a nonstructured literature search of SRs where we tried to find SR abstracts that were simple to understand and appropriate for the target population. The abstracts did not contain any mention of the study design used. If the abstract reported that it was an abstract of an SR or if a systematic search was mentioned, that part of the abstract was removed. The participants were asked to assess whether the abstracts were abstracts of SRs. The 4 abstracts used for this assessment are presented in Multimedia Appendix 6 [25-28].

On the last page of the interface in both intervention groups A and B, the participants were invited to optionally leave their first and last name and email address if they wanted to receive a certificate of participation in the educational intervention. The certificate was prepared by Cochrane Croatia.

The entire questionnaire we administered to the participants was a survey and not a psychological instrument. Thus, we did not perform any psychometric calculations. For the 6 before-and-after questions about the opinion regarding SRs, we calculated that, at the first measurement (before the intervention), reliability was .89, expressed using Cronbach *α*.

### Outcomes

The primary outcome was the difference in the percentage of correct answers per participant in the postintervention questionnaire between intervention groups A and B.

Secondary outcomes were the difference in the percentage of correct answers per participant in the pre- and postintervention questionnaires for the intervention group, the proportion of participants who correctly recognized an abstract describing an SR of the literature (percentage), and the proportion of participants who correctly recognized an abstract describing a simple narrative review of the literature (percentage).

### Participant Timeline

After we obtained permission from the ethics committees, the participants were invited to take part. After collecting the names of students who agreed to participate and randomizing them, the invitation to participate in the study containing the link to the intervention A or intervention B interface was sent on June 7, 2021. The links were inactivated on June 20, 2021. The knowledge assessment was conducted immediately after the intervention.

### Sample Size

The expected effect size was a difference of at least 20% for the primary outcome between intervention groups A and B. The calculation of the sample size to compare the proportions, predefining an *α* of .05 and *β* of .20, assuming a difference of at least 20% for the primary outcome between intervention groups A and B, determined that a sample size of 182 participants (91 participants per group) would be required. To compensate for the possible loss of participants after the beginning of the survey (incomplete answers) or the possibility that participants who initially agreed to take part might eventually choose not to take part, the plan was to include at least 20% more participants than calculated as necessary (n=218).

### Encouraging the Inclusion of Participants (Recruitment)

After the initial email was sent to the participants with the link to their respective study arm, 3 more reminders were sent to the participants 4 days apart.

### Randomization of Participants

The participants were randomized by simple randomization using the Randomizer website.

### Allocation Concealment

After randomization, the participants were allocated to the study arms using a randomization sequence by a third person who was not included in other parts of the study.

### Blinding

#### Blinding of Participants and Personnel

The intervention was of such a nature that the participants could not be blinded.

#### Blinding of Outcome Assessors

Only the first author (MKM) and the principal investigator (LP) had access to the complete raw data set generated by SurveyMonkey, which included the names and email addresses of the participants who wanted the certificate. MKM removed the participants’ names and email addresses before the outcome assessor (IB) analyzed the data; thus, anonymized data were analyzed.

### Data Management

One author downloaded Microsoft Excel worksheets from SurveyMonkey, which were anonymized in case any participant left a name and email address to obtain the certificate. The SurveyMonkey interface was configured not to collect any information about the participants, including IP addresses. The data were stored on a secure server until the time of analysis.

### Statistical Analysis

To determine the normality of the variables’ distribution, we used the Kolmogorov-Smirnov test. Categorical data were presented as frequencies and percentages, and numerical values were presented as medians with IQR for variables not following normal distribution and as arithmetic means with IQR for variables following normal distribution. Differences between intervention groups A and B for categorical variables were tested using the chi-square test. To express the difference between groups, numerical values were tested with 2-tailed *t* tests for independent samples (for variables following normal distribution) and Mann-Whitney tests (for variables not following normal distribution). Pre- and postintervention differences were evaluated using the chi-square test for categorical variables and the *t* test for independent samples for numerical variables. The effect size for the primary outcome (the difference between the percentage of correct answers between groups in the postintervention questionnaire) was expressed using relative risk (RR) and 95% CI, as was the difference between the number of correct answers in the pre- and postintervention questionnaires in both groups. The effect size for the secondary outcome was expressed using odds ratio with 95% CI.

We assessed the participants’ opinions before and after using parametric procedures on Likert-type scales, which are usually analyzed using a nonparametric test. This was done because, after the initial analysis where we used nonparametric statistics, the results were not interpretable. When we presented results using median and 95% CIs, the results were similar in both groups, although there were significant differences after the intervention. Therefore, we proceeded with parametric testing, which gave the same results but was more precise as it enabled us to interpret the direction of the difference clearly.

All analyses were performed using the computer program JASP (version 0.14.1.0; JASP Team). Statistical significance was set at *P*<.05.

## Results

### Participant Flow

In this trial, 871 potential participants met the inclusion criteria, of whom 282 (32.4%) indicated that they did not want to participate in the study. Thus, 67.6% (589/871) of students were randomized: 31.1% (183/589) from University North; 23.1% (136/589) from the Catholic University of Croatia; 22.4% (132/589) from the Faculty of Health Studies, University of Rijeka; 14.3% (84/589) from the University Department of Health Studies Split; 6.6% (39/589) from the University Department of Health Studies Zadar; 1.7% (10/589) from the University of Dubrovnik, Nursing Studies; and 0.8% (5/589) from the Faculty of Dental Medicine and Health, University of Osijek.

### Recruitment and Access to the Educational Platform

**Figure 1:**
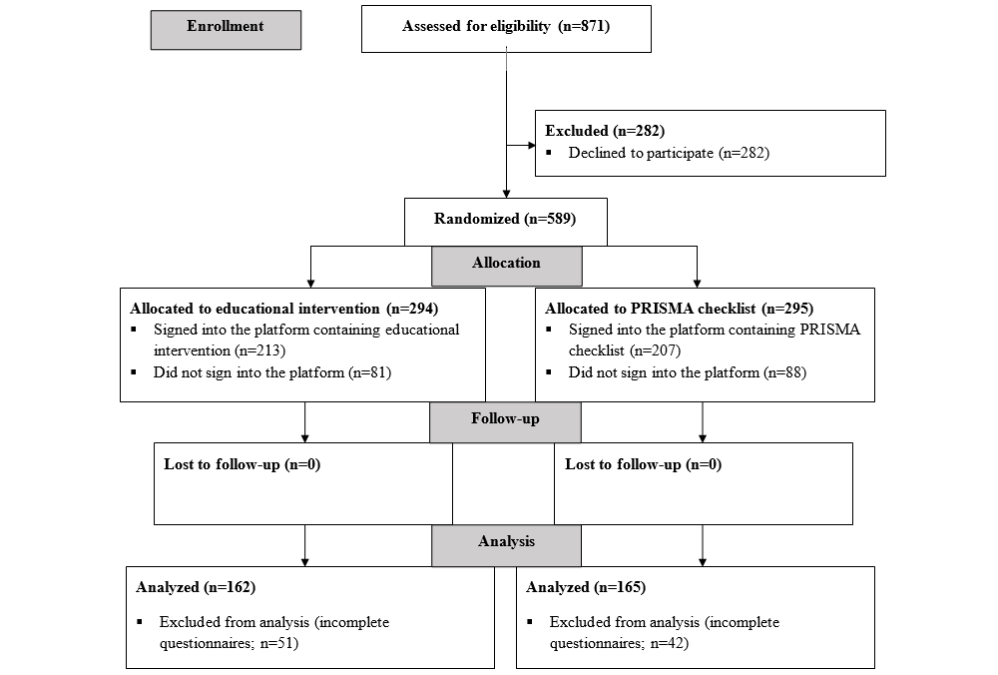
Participant flow diagram. PRISMA: Preferred Reporting Items for Systematic Reviews and Meta-Analyses.

The link to participate in the study was sent via email to the addresses of the 589 students on June 7, 2021. The students were sent 3 reminders 4 days apart, and access to the web-based platforms was inactivated on June 20, 2021. A detailed participant flow diagram is shown in Figure 1.

The average time the participants took to complete the entire interface with questionnaires and educational materials was 21 (SD 9.00) minutes in the educational intervention group and 19 (SD 3.96) minutes in the PRISMA checklist group.

### Baseline Participant Characteristics

The demographic participant data are presented in Table 1. More than 40% of the participants (educational intervention group: 66/162, 40.7%; PRISMA checklist group: 64/165, 38.8%) were from 1 institution (University North), >80% of the participants (educational intervention group: 134/162, 82.7%; PRISMA checklist group: 138/165, 83.6%) studied nursing, and >50% of the participants (educational intervention group: 97/162, 59.9%; PRISMA checklist group: 85/165, 51.5%) attended the second year of study. More than 80% of the participants (educational intervention group: 136/162, 84%; PRISMA checklist group: 138/165, 83.6%) were employed while studying for their Master’s degree. Most participants were employed as health care workers (educational intervention group: 130/162, 80.2%; PRISMA checklist group: 131/165, 79.4%). The median length of working in health care was 9.9 years among participants who received the educational intervention and 9.8 years in the PRISMA checklist group. The median age of the participants in both groups was approximately 30 years, and >85% of the participants in both groups were women (educational intervention group: 140/162, 86.4%; PRISMA checklist group: 146/165, 88.5%; Table 1).

Participants in both groups rated their knowledge of SRs with a median grade of 3 (range 1-5). All participants (327/327, 100%) stated that they had heard of SRs, and approximately three-quarters of the participants in both groups (educational intervention group: 124/162, 76.5%; PRISMA checklist group: 123/165, 74.5%) stated that they had read an SR. In our sample, 17.3% (28/162) of the participants from the group that received the educational intervention and 18.2% (30/165) of the participants from the PRISMA checklist group stated that they had participated in producing an SR (Table 1).

**Table 1:** Demographic characteristics of the participants included in the analysis (N=327).

Variable and level		Educational intervention (n=162)	PRISMA^a^ checklist (n=165)
**Institution, n (%)**			
	Faculty of Dental Medicine and Health Osijek	3 (1.9)	2 (1.2)
	Faculty of Health Studies, University of Rijeka	26 (16)	30 (18.2)
	Croatian Catholic University	46 (28.4)	41 (24.8)
	Health Department, University of Zadar	18 (11.1)	20 (12.1)
	Health Studies, University of Dubrovnik	0 (0)	3 (1.8)
	Health Studies, University of Split	3 (1.9)	5 (3)
	University North	66 (40.7)	64 (38.8)
**Study program, n (%)**			
	Physiotherapy	17 (10.5)	15 (9.1)
	Clinical Nutrition	5 (3.1)	8 (4.8)
	Radiological Technology	2 (1.2)	1 (0.6)
	Nursing	134 (82.7)	138 (83.6)
	Something else	4 (2.5)	3 (1.8)
**Year of study, n (%)**			
	First	59 (36.4)	74 (44.8)
	Second	97 (59.9)	85 (51.5)
	Third	6 (3.7)	6 (3.6)
Currently employed (yes), n (%)		136 (84)	138 (83.6)
Currently employed as a health care worker (yes), n (%)		130 (80.2)	131 (79.4)
Length of work status (years), median (IQR)		7 (3-15)	6 (2-16)
Age (years), median (IQR)		28 (24-35)	26 (24-34)
Women, n (%)		140 (86.4)	146 (88.5)
Self-assessment of knowledge of EBM^b^ (1-5), median (IQR)		3 (3-4)	3 (3-4)
Had heard about systematic reviews, n (%)		162 (100)	165 (100)
Had read a systematic review, n (%)		124 (76.5)	123 (74.5)
Had participated in writing a systematic review, n (%)		28 (17.3)	30 (18.2)

^a^PRISMA: Preferred Reporting Items for Systematic Reviews and Meta-Analyses.

^b^EBM: evidence-based medicine.

### Numbers Analyzed

Of the 420 participants who accessed the interface, 327 (77.9% response rate) completed the questionnaires, and their results were included in further analysis (Figure 1).

Owing to incomplete questionnaires, we excluded the results of 23.9% (51/213) of participants from the educational intervention group and 20.3% (42/207) of participants from the PRISMA checklist group. The results of 54.9% (162/295) of participants from the educational intervention group and 56.1% (165/294) of participants from the PRISMA checklist group were finally included in the analysis. There were no transfers of participants from one group to another.

### Primary Outcome

In the postintervention questionnaire, of the 1458 potential correct answers, there were 1086 (74.49%) correct answers to knowledge questions in the educational intervention group (162/327, 49.5%). In the PRISMA checklist group (165/327, 50.5%), of the 1485 potential correct answers, there were 900 (60.61%) correct answers (Table 2). Thus, the effect size for the difference in the number of correct answers to knowledge questions between groups was an RR of 1.23 (95% CI 1.17-1.29); that is, the educational intervention group had 23% (relative risk percentage) more correct answers in the postintervention questionnaire than the PRISMA checklist group.

**Table 2:** Knowledge of systematic reviews (SRs) among participants who completed pre- and postintervention assessments (N=327).

Questionnaire and items		Educational intervention (n=162)	PRISMA^a^ checklist (n=165)	*P* value^b^
**Preintervention questionnaire (correct answer)**				
	It is sufficient to search one database to produce an SR (no), n (%)	128 (79)	139 (84.2)	.22
	SRs must be produced by one author only (no), n (%)	103 (63.6)	98 (59.4)	.44
	SRs must contain meta-analyses (no), n (%)	17 (10.5)	21 (12.7)	.52
	SRs must have duplicate screening and data extraction (yes), n (%)	87 (53.7)	83 (50.3)	.54
	A list of both included and excluded studies must be provided (yes), n (%)	117 (72.2)	116 (70.3)	.70
	The quality of the included studies must be assessed (yes), n (%)	135 (83.3)	143 (86.7)	.40
	In the case of meta-analyses, a heterogeneity test must be done to ensure the results of the studies can be combined (yes), n (%)	126 (77.8)	126 (76.4)	.76
	Results of meta-analyses must be presented as a funnel plot (no), n (%)	31 (19.1)	13 (7.9)	.003
	Results of publication bias analysis must be presented as a forest plot (no), n (%)	31 (19.1)	27 (16.4)	.51
	Total correct answer scores, mean (95% CI)	4.8 (4.5-5.0)	4.6 (4.4-4.9)	.44
**Postintervention questionnaire (correct answer)**				
	It is sufficient to search one database to produce an SR (no), n (%)	156 (96.3)	120 (72.7)^c^	<.001
	SRs must be produced by one author only (no), n (%)	153 (94.4)	126 (76.4)	<.001
	SRs must contain meta-analyses (no), n (%)	38 (23.5)^c^	33 (20)^c^	.45
	SRs must have duplicate screening and data extraction (yes), n (%)	144 (88.9)	111 (67.3)^c^	<.001
	A list of both included and excluded studies must be provided (yes), n (%)	144 (88.9)	141 (85.5)^c^	.35
	The quality of the included studies must be assessed (yes), n (%)	155 (95.7)^c^	142 (86.1)	.003
	In the case of meta-analyses, a heterogeneity test must be done to ensure the results of the studies can be combined (yes), n (%)	150 (92.6)^c^	146 (88.5)^c^	.20
	Results of meta-analyses must be presented as a funnel plot (no), n (%)	80 (49.4)^c^	44 (26.7)^c^	<.001
	Results of publication bias analysis must be presented as a forest plot (no), n (%)	66 (40.7)^c^	37 (22.4)^c^	<.001
	Total correct answer scores, mean (95% CI)	6.7 (6.5-6.9)^c^	5.5 (5.3-5.7)^c^	<.001

^a^PRISMA: Preferred Reporting Items for Systematic Reviews and Meta-Analyses.

^b^Comparison between educational intervention and PRISMA checklist groups. Chi-square test was used for categorical variables, and 2-tailed *t* test was used for independent samples for numeric variables.

^c^Comparison before and after the intervention. Chi-square test was used for categorical variables, and 2-tailed *t* test was used for dependent samples for numeric variables.

### Secondary Outcomes

#### Difference in the Number of Correct Answers per Participant in the Pre- and Postintervention Questionnaires for the Educational Intervention Group

Both groups performed better on the postintervention questionnaire than on the preintervention questionnaire (Table 2). In the educational intervention group, the total number of correct answers was 53.16% (775/1458) in the preintervention questionnaire and 74.49% (1086/1458) in the postintervention questionnaire (RR=1.40, 95% CI 1.32-1.48; Table 2). In the PRISMA checklist group, the total number of correct answers was 51.58% (766/1485) in the preintervention questionnaire and 60.61% (900/1485) in the postintervention questionnaire (RR=1.17, 95% CI 1.10-1.25; Table 2).

Independent of the group, in the pre- and postintervention questionnaires, the smallest number of correct answers was to questions related to the concept of meta-analysis, whereas, in both groups, the highest number of correct answers was to the question about the necessity to assess the quality of research included in the SR (Table 2).

There was no difference in the overall results of the questionnaire assessing knowledge of SRs (the exact number of answers to all 9 knowledge questions) between the educational intervention and PRISMA checklist groups before the intervention (Table 2).

#### Proportion of Participants Who Correctly Recognized SR Abstracts

The first 2 presented summaries were identified accurately as summaries of SRs by 65.4% (106/162) and 74.1% (120/162) of participants from the educational intervention group and 71.5% (118/165) and 72.7% (120/165) of participants in the PRISMA checklist group, respectively (Table 3). There was no statistically significant difference between the groups in the ability to correctly detect an SR summary (Table 3).

The third and fourth summaries were recognized as a summary of a simple narrative review by 22.2% (36/162) and 46.3% (75/162) of participants from the educational intervention group and 34.5% (57/165) and 47.9% (79/165) of participants from the PRISMA checklist group, respectively (Table 3). There was no statistically significant difference between the groups in the recognition of summaries of narrative reviews (Table 3).

**Table 3:** Comparison of answers to questions on sources of information needed to answer a clinical question and recognition of a systematic review (SR) abstract (N=327).

Variable and level			Educational intervention (n=162), n (%)		PRISMA^a^ checklist (n=165), n (%)		*P* value^b^
**If you needed to search for information to solve a clinical problem, what would be the preferred information source for you?**							
	Colleagues	65 (40.1)		65 (39.4)		.98	
	Books	59 (36.4)		61 (37)		.98	
	Scientific literature	120 (74.1)		132 (80)		.25	
	SR of the literature	135 (83.3)		136 (82.4)		.93	
	Internet search engine (Google)	29 (17.9)		30 (18.2)		.98	
**Is this an SR abstract?**							
	Abstract 1^c^—correct answer “Yes”	106 (65.4)		118 (71.5)		.34	
	Abstract 2^d^—correct answer “Yes”	120 (74.1)		120 (72.7)		.56	
	Abstract 3^e^—correct answer “No”	36 (22.2)		57 (34.5)		.02	
	Abstract 4^f^—correct answer “No”	75 (46.3)		79 (47.9)		.78	

^a^PRISMA: Preferred Reporting Items for Systematic Reviews and Meta-Analyses.

^b^Chi-square test.

^c^A total of 7 answers missing.

^d^A total of 7 answers missing.

^e^A total of 8 answers missing.

^f^A total of 10 answers missing.

#### Additional Analyses

There was no statistical difference in the choice of information sources between the educational intervention and PRISMA checklist groups in the postintervention questionnaire, with multiple possible responses about where the participants would look for answers to a clinical question from their own clinical practice (Table 3). More than 80% of the participants in both groups (educational intervention group: 135/162, 83.3%; PRISMA checklist group: 136/165, 82.4%) stated that they would look for answers in an SR. Most participants (educational intervention group: 120/162, 74.1%; PRISMA checklist group: 132/165, 80%) responded that they would look for answers in scientific literature in general (Table 3). A third of the participants in both groups would look for an answer to a clinical question in a textbook (educational intervention group: 59/162, 36.4%; PRISMA checklist group: 61/165, 37%) or ask a coworker for an answer (educational intervention group: 65/162, 40.1%; PRISMA checklist group: 65/165, 39.4%). Less than one-fifth of the participants in both groups would search for an answer on an internet search engine such as Google (educational intervention group: 29/162, 17.9%; PRISMA checklist group: 30/165, 18.2%; Table 3).

In the preintervention assessment in both groups of participants, there was no significant difference in agreement with the proposed characteristics of an SR (Table 4). After the intervention, there was more agreement with these characteristics in the educational intervention group than in the PRISMA checklist group (Table 4).

**Table 4:** Responses regarding the characteristics of a systematic review in the pre- and postintervention questionnaires (N=327)^a^.

Questionnaire and items		Educational intervention (n=162), mean (95% CI)	PRISMA^b^ checklist (n=165), mean (95% CI)	*P* value^c^
**Preintervention questionnaire**				
	Research question is defined	4.5 (4.4-4.7)	4.4 (4.2-4.5)	.09
	Listed sources of literature searched, with repeatable search strategy (naming of databases, naming of search platforms, search date, and complete search strategy)	4.4 (4.2-4.5)	4.2 (4.0-4.3)	.08
	Listed criteria for inclusion and exclusion of research	4.5 (4.3-4.6)	4.3 (4.1-4.4)	.04
	Listed selection methods	4.4 (4.3-4.6)	4.3 (4.2-4.5)	.45
	Critically evaluates and reports on the quality or risk of bias of the included studies	4.4 (4.2-4.5)	4.1 (4.0-4.3)	.04
	Provides information on data analysis and synthesis that allows for the repeatability of the results	4.4 (4.2-4.5)	4.2 (4.1-4.4)	.11
**Postintervention questionnaire**				
	Research question is defined	4.8 (4.7-4.9)	4.6 (4.5-4.7)	<.001
	Listed sources of literature searched, with repeatable search strategy (naming of databases, naming of search platforms, search date, and complete search strategy)	4.7 (4.6-4.8)	4.6 (4.5-4.7)	.05
	Listed criteria for inclusion and exclusion of research	4.8 (4.7-4.9)	4.5 (4.4-4.6)	<.001
	Listed selection methods	4.8 (4.7-4.9)	4.6 (4.5-4.7)	.02
	Critically evaluates and reports on the quality or risk of bias of the included studies	4.7 (4.6-4.8)	4.5 (4.4-4.6)	<.001
	Provides information on data analysis and synthesis that allows for the repeatability of the results	4.7 (4.6-4.8)	4.5 (4.3-4.6)	<.001

^a^All differences before and after were statistically significant at *P*<.05; 2-tailed *t* test for paired samples.

^b^PRISMA: Preferred Reporting Items for Systematic Reviews and Meta-Analyses.

^c^*t* test (2-tailed) for independent samples.

## Discussion

### Principal Findings

This RCT demonstrated that a brief educational intervention conducted on the web about SRs significantly increased knowledge of SRs in the target population. To the best of our knowledge, this is the first trial conducted for this purpose. Relatively successful learning models about EBM have been reported in the literature [15,29-31], but we could not find any publications on the effectiveness of educational interventions focused exclusively on knowledge of SRs.

### Comparison With Prior Work

The participants from the educational intervention group (162/327, 49.5%), who were presented with a new educational intervention designed for this study, needed an average of 21 minutes to go through the entire interface. The interface included multiple sections beyond educational intervention: pre- and postintervention questionnaires and the evaluation of 4 scientific abstracts. However, the web-based platform used for this study did not allow for the measurement of the time spent on specific items or pages in the interface. Thus, we cannot know how long the participants read the educational texts prepared for the educational intervention and PRISMA checklist groups. However, if we consider the time to read and answer the questions, the participants probably needed 15 minutes or less to read the educational intervention itself. Such an intervention is very short. Therefore, the intervention should be suitable for health professionals who usually state that their lack of time is a major obstacle to practicing EBM [32-34] and implementing the EBM curriculum during education [35].

Initially, participants in both groups rated their knowledge of SRs with a median grade of 3 out of 5. This is comparable with the self-assessed knowledge of EBP among nurses evaluated in the study conducted by Munroe et al [6]. In that study, only 3% of the nurses said that they were very familiar with EBP [6].

Three-quarters of the participants (educational intervention group: 124/162, 76.5%; PRISMA checklist group: 123/165, 74.5%) stated that they had read SRs. We were surprised with the result that 17.3% (28/162) and 18.2% (30/165) of the participants in the educational intervention and PRISMA checklist groups, respectively, stated that they had participated in developing a SR, which is a high percentage [6]. SR methodology is very complex. Thus, it is questionable whether the students have actually participated in the development of SRs in such large numbers. Health students may have participated, for example, in translating Cochrane’s plain language summaries into Croatian [36,37]. However, without the possibility of further clarifying what the participants really meant, it is not possible to discuss this topic in further detail. In the study by Olsson et al [38], which focused on nursing PhD programs and candidatures, in the analyzed 135 nursing dissertations made according to the Scandinavian model of integrated research, only 5 published SRs were found (ie, only 4% of the included nurses—dissertation authors—participated in developing an SR). This number is much lower than the percentage of our participants who stated that they had participated in the production of an SR, and our students were not PhD students but Master’s-level students.

The primary outcome of this study was the difference in the percentage of correct answers collected from the educational intervention and PRISMA checklist groups when answering questions evaluating knowledge of SRs on the postintervention questionnaire after the participants had read the educational materials. After the training, the educational intervention group had 23% more correct answers than the PRISMA checklist group (ie, the size of the effect expressed in RR was 1.23). In addition, comparing the pre- and postintervention questionnaire results in the educational intervention group, there were significantly more correct answers on the postintervention questionnaire than on the preintervention questionnaire, with an RR of 1.40. The RR of correct answers comparing the pre- and postintervention questionnaires in the PRISMA checklist group was 1.17.

An RCT by Sánchez-Mendiola et al [15] showed a significant effect of EBM education on the final knowledge of EBM among medical students, with a 25.9% increase in correct answers in the knowledge test about EBM [15]. This is comparable with our primary outcome results. However, it should be emphasized that their intervention was very different in terms of content and duration. Sánchez-Mendiola et al [15] tested an EBM course with 14 two-hour weekly sessions during 1 semester. The course was a formal part of the medical school curriculum; it was delivered by 6 experienced professors and included different content compared with ours. Their course covered 15 topics, including clinical decision-making, uncertainty and probability in medicine, the Bayes theorem, and clinical guidelines [15].

Rohwer et al [18] evaluated the effectiveness of e-learning in improving EBHC in a Campbell SR. The study included 24 trials, of which 20 were RCTs and 4 were observational studies, with a total of 3825 participants including physicians, nurses, physiotherapists, physician assistants, and educators at all levels of education. It demonstrated that, compared with nonlearning, pure e-learning improved EBHC knowledge and skills with similar outcomes to face-to-face learning for any observed primary outcome.

In 2021, needs assessments and expectations regarding EBP knowledge acquisition and training activities were explored among frontline health care providers, including postgraduate medical and nursing students who were working or living in China. The results indicated that the respondents expressed a high need for education on evidence quality appraisal, interpretation of SRs or meta-analyses, and knowledge translation [39]. However, it may not be sufficient to only strive for the improvement of knowledge among the targeted individuals. Nursing education at the undergraduate level is starting to teach the process of research integration through EBP implementation with active learning strategies, which is endorsed by the students [40].

To advance the knowledge and application of evidence in daily practice, ultimately, health institutions will also need to recognize the need to foster such topics [41].

Our educational intervention, implemented via a web tool, is particularly suitable in the current time of the COVID-19 pandemic. Owing to containment measures, many parts of the world have switched to web-based education during the COVID-19 pandemic. Bond et al [42] published a living systematic mapping review on August 30, 2021, calling the web-based teaching experience during the pandemic the “first global online semester.” Although such teaching was initially seen as a distance learning response to emergency remote teaching [43], the educational experience gained during a pandemic is very valuable for evaluating the distance learning experience. Our study provides a further test of a remotely delivered educational intervention targeting students and health care workers.

Many studies have evaluated experiences with virtual continuing medical education during the pandemic [44-48]. The SR education evaluated in our study could be incorporated into continuing medical education programs for health care professionals. Owing to the short format and the possibility of distance learning, such education could be of interest to health care professionals who want to learn more about the basics of SRs.

In addition to showing the efficacy of our newly designed educational intervention, our study also indicated areas where the target group of participants significantly lacked knowledge. Independent of the group, in the pre- and postintervention questionnaires, the smallest number of correct answers was to questions related to the concept of meta-analysis and questions about graphical representations of meta-analyses (funnel plot and forest plot). Very modest improvements were observed in those questions in the postintervention questionnaire. In a study on knowledge of the basic methodological components of SRs conducted by Puljak and Sapunar [23] among the directors of postgraduate programs at European universities, only 31% of the participants answered correctly that an SR does not necessarily contain a meta-analysis.

There were few correct answers to questions about graphical presentations of meta-analyses. In the educational intervention group, before the training, 20% of the participants correctly answered what a funnel plot and a forest plot represented. In the PRISMA checklist group, only 8% of the participants correctly answered what a funnel plot represented, and 16% correctly answered what a forest plot depicted. In the postintervention questionnaire, in the educational intervention group, 40.7% (66/162) to 49.4% (80/162) of the participants correctly answered the question about the use of the forest plot or funnel plot, whereas, in the PRISMA checklist group, only a fifth (37/165, 22.4%) to a quarter (44/165, 26.7%) of the participants correctly answered these questions. However, after the educational intervention, more than half of the participants did not know the correct answer to the questions regarding the graphical representations of meta-analyses.

Poor knowledge of graphical representations in meta-analyses has been described elsewhere. A survey conducted on psychologists in Italy found that less than a fifth of psychologists estimated that they had sufficient knowledge of the forest plot [49]. Less than 15% of psychologists stated that they had sufficient knowledge of the funnel plot [49]. A survey conducted on psychologists in Spain showed that only approximately 10% of the participants said that they had satisfactory knowledge of the forest plot. Only 7% of the participants said that they had satisfactory knowledge of the funnel plot [50]. Only 10% of PhD program directors accurately recognized the purpose of funnel plots, and 11.3% recognized the purpose of the forest plot [23]. Poor knowledge of graphical representations of meta-analyses may be the best indicator of generally poor knowledge of SR and meta-analysis methodology.

In this study, we also included a practical knowledge test that involved the recognition of journal abstracts of SRs after the intervention. The accuracy of journal abstract recognition was 22% to 72% in the educational intervention group and 46% to 74% in the PRISMA checklist group, without a statistically significant difference between the groups. This was the final test of understanding and pragmatic application of the knowledge acquired in our trial. We found that the educational intervention did not significantly affect the recognition of abstracts of SRs. It is possible that a time lag or longer systematic learning is needed for the acquired knowledge of SRs to influence the practical application of the knowledge itself. It is also possible that it is necessary to further adjust the educational intervention to enable the practical application of the acquired knowledge.

In the postintervention questionnaire, the participants were asked which sources of information they would use in searching for an answer to a question from their clinical practice, and most participants from both groups opted for scientific literature (educational intervention group: 120/162, 74.1%; PRISMA checklist group: 132/165, 80%) or SRs (educational intervention group: 135/162, 83.3%; PRISMA checklist group: 136/165, 82.4%). Compared with the results of a study conducted by Sánchez-Mendiola et al [15] on medical students in Mexico, in our study, a significantly higher percentage of students chose to search for answers in SRs and scientific literature. In the study by Sánchez-Mendiola et al [15], most participants from the group that attended EBM classes stated that, in solving a certain health problem, they looked for answers in review articles or the Cochrane Library only occasionally, whereas very often they would look for the answer to a health problem in textbooks or search engines or they would ask their teachers. Nevertheless, the number of students who would seek answers in the Cochrane Library or scientific articles was higher than in groups of students who did not attend classes on EBM [15]. Evidently, education about EBM—or, in our case, about SRs—has the express intention to use these data sources more often in solving clinical problems.

### Strengths and Limitations

The strength of this study is the appropriate sample size and a high number of fully completed questionnaires, with three-quarters of the participants (327/420, 77.9%) completing the questionnaire in full and almost equal numbers of unfinished questionnaires in the educational intervention and PRISMA checklist groups, allowing for comparable results. Furthermore, we tested the practical application of the acquired knowledge of SRs by asking the students to recognize summaries of SRs or narrative reviews.

A limitation of this study is a highly homogeneous sample that does not allow for significant analyses by sociodemographic subgroups. We acknowledge that there is a potential self-selection aspect in our final sample. We do not have data about nonparticipants among the eligible students and, theoretically, there could be some differences between responders and nonresponders. However, this is an inherent problem of any trial—the eligible participants are invited to take part, and they can choose whether they want to participate.

The study was conducted in only 1 country but in multiple institutions across the country. We did not measure the time spent in the intervention; some participants may not have spent much time reading the text. Furthermore, we measured the outcomes immediately after the reading of the educational texts. Such short-term follow-up does not allow for monitoring of the long-term retention of knowledge of SRs among the participants. Longer-term research will make it possible to verify the long-term effectiveness of the intervention on the knowledge of the target group.

Finally, we would like to note that, in this manuscript, when referring to the studies of other researchers, we used the terms EBM, EBP, and EBHC as they were reported in those manuscripts.

### Conclusions

A short web-based educational intervention about SRs is an effective tool for short-term improvement of knowledge of SRs among health care studies students, most of whom were employed as health care professionals. This education can be further studied, modified, and used in the continuing medical education of health care professionals.

## Appendix

Multimedia Appendix 1 CONSORT-eHEALTH checklist (V 1.6.2).

Multimedia Appendix 2 Text of the first email and reminders to invite participants to a randomized controlled trial.

Multimedia Appendix 3 Educational intervention.

Multimedia Appendix 4 PRISMA (Preferred Reporting Items for Systematic Reviews and Meta-Analyses) 2009 checklist (divided into 11 sections for the purpose of the study).

Multimedia Appendix 5 The text of the pre- and postintervention questionnaires.

Multimedia Appendix 6 Four abstracts selected for assessment.

## Abbreviations

CONSORT: Consolidated Standards of Reporting Trials
EBHC: evidence-based health care
EBM: evidence-based medicine
EBP: evidence-based practice
GREET: Guideline for Reporting Evidence-based Practice Educational Interventions and Teaching
PRISMA: Preferred Reporting Items for Systematic Reviews and Meta-Analyses
RCT: randomized controlled trial
RR: relative risk
SR: systematic review

## Data Availability

The raw data from this study were published on the Open Science Framework website [21] and made publicly available.
