# Supplementary material for: Web-Based Educational Intervention to Improve Knowledge of Systematic Reviews Among Health Science Professionals: Randomized Controlled Trial
Source: J Med Internet Res. 2022 Aug 25;24(8):e37000. doi: 10.2196/37000 (PMC9459937; doi:10.2196/37000)
Supplement: Multimedia Appendix 1 [file jmir_v24i8e37000_app1.pdf]

CONSORT-EHEALTH Checklist V1.6.2 Report	Manuscript Number	37000

(based on CONSORT-EHEALTH V1.6), available at [http://tinyurl.com/consort-ehealth-v1-6].

Date completed
8/7/2022 13:13:28
by
Marina Krnic

Web-Based Educational Intervention to Improve Knowledge of
Systematic Reviews Among Health Science Professionals:
Randomized Controlled Trial

TITLE

1a-i) Identify the mode of delivery in the title
"Web-Based Educational Intervention to Improve Knowledge"
1a-ii) Non-web-based components or important co-interventions in title
No, this intervention was completely web-based.
1a-iii) Primary condition or target group in the title
"Among Health Science Professionals"

ABSTRACT

1b-i) Key features/functionalities/components of the intervention and comparator in the METHODS section of the ABSTRACT
"Intervention group A (294/589, 49.9%) received a short web-based educational intervention about SR methodology, and intervention group B (295/589, 50.1%) was presented with the PRISMA (Preferred Reporting Items for Systematic Reviews and Meta-Analyses) checklist."
1b-ii) Level of human involvement in the METHODS section of the ABSTRACT

1b-iii) Open vs. closed, web-based (self-assessment) vs. face-to-face assessments in the METHODS section of the ABSTRACT

1b-iv) RESULTS section in abstract must contain use data

1b-v) CONCLUSIONS/DISCUSSION in abstract for negative trials

INTRODUCTION

2a-i) Problem and the type of system/solution
"Knowledge of SRs in trainees can help not only in developing useful skills in critical appraisal but also in addressing important clinical questions and serve as a strong basis to design new, original research studies that will fill the gaps and answer relevant and unsolved clinical questions."
2a-ii) Scientific background, rationale: What is known about the (type of) system
" It has been shown that better knowledge and more positive attitudes toward EBM among medical students are associated
with the exposure to the vertical subject on research in biomedicine and activities of The Cochrane Collaboration."

Does your paper address CONSORT subitem 2b?
"In this randomized controlled trial (RCT), we evaluated the effect of a short web-based educational intervention about SRs on short-term knowledge of SR among students of health sciences studies in Croatia."

METHODS

3a) CONSORT: Description of trial design (such as parallel, factorial) including allocation ratio
"We conducted an RCT with 2 parallel groups and 1:1 participant allocation."
3b) CONSORT: Important changes to methods after trial commencement (such as eligibility criteria), with reasons
There were no important changes to methods after trial commencement.
3b-i) Bug fixes, Downtimes, Content Changes

4a) CONSORT: Eligibility criteria for participants
"The participants were students of Master’s university health sciences studies in Croatia."
4a-i) Computer / Internet literacy

4a-ii) Open vs. closed, web-based vs. face-to-face assessments:
"Students from eligible institutions were contacted via email by coauthors (MC, MN, KI, DA, NS, SZ, and SM) employed in these institutions and invited to participate in the study on brief web-based education about SRs of the literature."
4a-iii) Information giving during recruitment

4b) CONSORT: Settings and locations where the data were collected
"There were 8 eligible institutions in Croatia for this study, and we invited all of them."
4b-i) Report if outcomes were (self-)assessed through online questionnaires
Outcomes were (self-)assessed through online questionnaires.
4b-ii) Report how institutional affiliations are displayed

5) CONSORT: Describe the interventions for each group with sufficient details to allow replication, including how and when they were actually administered
5-i) Mention names, credential, affiliations of the developers, sponsors, and owners

5-ii) Describe the history/development process

5-iii) Revisions and updating

5-iv) Quality assurance methods

5-v) Ensure replicability by publishing the source code, and/or providing screenshots/screen-capture video, and/or providing flowcharts of the algorithms used

5-vi) Digital preservation

5-vii) Access
"For this study, 2 separate interfaces for the participants were created on the SurveyMonkey platform (Momentive Inc)."
5-viii) Mode of delivery, features/functionalities/components of the intervention and comparator, and the theoretical framework
"Intervention Group A
In the web-based interface, intervention group A received a newly developed intervention created by the authors of this study with expertise in medical and health sciences education
and research methodology. The educational intervention was written in the Croatian language. It consisted of 11 short educational texts on the methodology of producing SRs.

Intervention Group B
Intervention group B was presented with the PRISMA checklist [22] for reporting on SRs (Multimedia Appendix 4) in their web-based interface, and the participants were asked to read it. It was presented to the participants in 11 separate sections to be as similar as possible to the number and form of the educational texts in intervention group A."
5-ix) Describe use parameters

5-x) Clarify the level of human involvement

5-xi) Report any prompts/reminders used
"After the initial email was sent to the participants with the link to their respective study arm, 3 more reminders were sent to the participants 4 days apart."
5-xii) Describe any co-interventions (incl. training/support)

"There were no conducted co-interventions."		
**6a) CONSORT: Completely defined pre-specified primary and secondary outcome measures, including how and when they were assessed**		
"The primary outcome was the difference in the percentage of correct answers per participant in the postintervention questionnaire between intervention groups A and B.<br><br>Secondary Outcomes were the difference in the number of correct answers per participant in the pre- and postintervention questionnaires for the educational intervention group and the proportion of participants who correctly recognized SR abstracts"		
**6a-i) Online questionnaires: describe if they were validated for online use and apply CHERRIES items to describe how the questionnaires were designed/deployed**		
**6a-ii) Describe whether and how "use" (including intensity of use/dosage) was defined/measured/monitored**		
**6a-iii) Describe whether, how, and when qualitative feedback from participants was obtained**		
**6b) CONSORT: Any changes to trial outcomes after the trial commenced, with reasons**		
"There were 8 eligible institutions in Croatia for this study, and we invited all of them."		
**7a) CONSORT: How sample size was determined**		
**7a-i) Describe whether and how expected attrition was taken into account when calculating the sample size**		
**7b) CONSORT: When applicable, explanation of any interim analyses and stopping guidelines**		
"The primary outcome was the difference in the percentage of correct answers per participant in the postintervention questionnaire between intervention groups A and B.<br><br>Secondary Outcomes were the difference in the number of correct answers per participant in the pre- and postintervention questionnaires for the educational intervention group and the proportion of participants who correctly recognized SR abstracts"		
**8a) CONSORT: Method used to generate the random allocation sequence**		
"The participants were randomized by simple randomization using the Randomizer website.<br>After randomization, the participants were allocated to the study arms using a randomization sequence by a third person who was not included in other parts of the study."		
**8b) CONSORT: Type of randomisation; details of any restriction (such as blocking and block size)**		
"The participants were randomized by simple randomization using the Randomizer website."		
**9) CONSORT: Mechanism used to implement the random allocation sequence (such as sequentially numbered containers), describing any steps taken to conceal the sequence until interventions were assigned**		
"After randomization, the participants were allocated to the study arms using a randomization sequence by a third person who was not included in other parts of the study."		
**10) CONSORT: Who generated the random allocation sequence, who enrolled participants, and who assigned participants to interventions**		
"After randomization, the participants were allocated to the study arms using a randomization sequence by a third person who was not included in other parts of the study."		
**11a) CONSORT: Blinding - If done, who was blinded after assignment to interventions (for example, participants, care providers, those assessing outcomes) and how**		
**11a-i) Specify who was blinded, and who wasn't**		
"The intervention was of such a nature that the participants could not be blinded.<br><br>MKM removed the participants'names and email addresses before the outcome assessor (IB) analyzed the data; thus, anonymized data were analyzed."		
**11a-ii) Discuss e.g., whether participants knew which intervention was the "intervention of interest" and which one was the "comparator"**		
**11b) CONSORT: If relevant, description of the similarity of interventions**		
Not relevant in this manuscript.		
**12a) CONSORT: Statistical methods used to compare groups for primary and secondary outcomes**		
"To determine the normality of the variables' distribution, we used the Kolmogorov-Smirnov test. Categorical data were presented as frequencies and percentages, and numerical values were presented as medians with IQR for variables not following normal distribution and as arithmetic means with IQR for variables following normal distribution. Differences between intervention groups A and B for categorical variables were tested using the chi-square test. To express the difference between groups, numerical values were tested with 2-tailed t tests for independent samples (for variables following normal distribution) and Mann-Whitney tests (for variables not following normal distribution). Pre- and postintervention differences were evaluated using the chi-square test for categorical variables and the t test for independent samples for numerical variables. The effect size for the primary outcome (the difference between the percentage of correct answers between groups in the postintervention questionnaire) was expressed using relative risk (RR) and 95% CI, as was the difference between the number of correct answers in the pre- and postintervention questionnaires in both groups. The effect size for the secondary outcome was expressed using odds ratio with 95% CI."		
**12a-i) Imputation techniques to deal with attrition / missing values**		
Not applicable to our manuscript.		
**12b) CONSORT: Methods for additional analyses, such as subgroup analyses and adjusted analyses**		
Not applicable to this manuscript.		
RESULTS		
**13a) CONSORT: For each group, the numbers of participants who were randomly assigned, received intended treatment, and were analysed for the primary outcome**		
A total of 294 participants were assigned to the intervention A group and 295 participants were assigned to the intervention B group.		
**13b) CONSORT: For each group, losses and exclusions after randomisation, together with reasons**		
A total of 81 participants form the group A and 88 participants from the group B were lost after randomization due to not signing into the platform.		
**13b-i) Attrition diagram**		
**14a) CONSORT: Dates defining the periods of recruitment and follow-up**		
"The link to participate in the study was sent via email to the addresses of the 589 students on June 7, 2021. The students were sent 3 reminders 4 days apart, and access to the web-based platforms was inactivated on June 20, 2021."		
**14a-i) Indicate if critical "secular events" fell into the study period**		
**14b) CONSORT: Why the trial ended or was stopped (early)**		
The trial did not end or was not stopped early.		
**15) CONSORT: A table showing baseline demographic and clinical characteristics for each group**		
The demographic characteristics are presented in Table 1 of the manuscript.		
**15-i) Report demographics associated with digital divide issues**		
"The median length of working in health care was 9.9 years among participants who received the educational intervention and 9.8 years in the PRISMA checklist group. The median age of the participants in both groups was approximately 30 years, and<br>>85% of the participants in both groups were women (educational intervention group: 140/162, 86.4%; PRISMA checklist group: 146/165, 88.5%;."		
**16a) CONSORT: For each group, number of participants (denominator) included in each analysis and whether the analysis was by original assigned groups**		
**16-i) Report multiple "denominators" and provide definitions**		
"In the postintervention questionnaire, of the 1458 potential correct answers, there were 1086 (74.49%) correct answers to knowledge questions in the educational intervention group (162/327, 49.5%). In the PRISMA checklist group (165/327, 50.5%), of the 1485 potential correct answers, there were 900 (60.61%) correct answers (Table 2). Thus, the effect size for the difference in the number of correct answers to knowledge<br>questions between groups was an RR of 1.23 (95% CI 1.17-1.29); that is, the educational intervention group had 23% (relative risk percentage) more correct answers in the<br>postintervention questionnaire than the PRISMA checklist group."		
**16-ii) Primary analysis should be intent-to-treat**		
**17a) CONSORT: For each primary and secondary outcome, results for each group, and the estimated effect size and its precision (such as 95% confidence interval)**		

"Thus, the effect size for<br>the difference in the number of correct answers to knowledge questions between groups was an RR of 1.23 (95% CI 1.17-1.29).<br><br>In the educational intervention group, the total number of correct answers was 53.16% (775/1458) in the preintervention questionnaire and 74.49% (1086/1458) in the postintervention questionnaire (RR=1.40, 95% CI 1.32-1.48; Table 2). In the PRISMA checklist group, the total number of correct answers was 51.58% (766/1485) in the preintervention questionnaire and 60.61% (900/1485) in the postintervention questionnaire<br>(RR=1.17, 95% CI 1.10-1.25)."		
**17a-i) Presentation of process outcomes such as metrics of use and intensity of use**		
**17b) CONSORT: For binary outcomes, presentation of both absolute and relative effect sizes is recommended**		
"In the postintervention questionnaire, of the 1458 potential correct answers, there were 1086 (74.49%) correct answers to knowledge questions in the educational intervention group (162/327, 49.5%). In the PRISMA checklist group (165/327, 50.5%), of the 1485 potential correct answers, there were 900 (60.61%) correct answers (Table 2). Thus, the effect size for the difference in the number of correct answers to knowledge<br>questions between groups was an RR of 1.23 (95% CI 1.17-1.29)."		
**18) CONSORT: Results of any other analyses performed, including subgroup analyses and adjusted analyses, distinguishing pre-specified from exploratory**		
"There was no statistical difference in the choice of information sources between the educational intervention and PRISMA checklist groups in the postintervention questionnaire, with multiple possible responses about where the participants would look for answers to a clinical question from their own clinical practice (Table 3)."		
**18-i) Subgroup analysis of comparing only users**		
**19) CONSORT: All important harms or unintended effects in each group**		
There were no reported harms or unintended effects.		
**19-i) Include privacy breaches, technical problems**		
**19-ii) Include qualitative feedback from participants or observations from staff/researchers**		
DISCUSSION		
**20) CONSORT: Trial limitations, addressing sources of potential bias, imprecision, multiplicity of analyses**		
**20-i) Typical limitations in ehealth trials**		
"A limitation of this study is a highly homogeneous sample that does not allow for significant analyses by sociodemographic subgroups. We acknowledge that there is a potential self-selection aspect in our final sample. We do not have data about nonparticipants among the eligible students and, theoretically, there could be some differences between responders and nonresponders. However, this is an inherent<br>problem of any trial—the eligible participants are invited to take part, and they can choose whether they want to participate."		
**21) CONSORT: Generalisability (external validity, applicability) of the trial findings**		
**21-i) Generalizability to other populations**		
**21-ii) Discuss if there were elements in the RCT that would be different in a routine application setting**		
**22) CONSORT: Interpretation consistent with results, balancing benefits and harms, and considering other relevant evidence**		
**22-i) Restate study questions and summarize the answers suggested by the data, starting with primary outcomes and process outcomes (use)**		
"The primary outcome of this study was the difference in the percentage of correct answers collected from the educational intervention and PRISMA checklist groups when answering<br>questions evaluating knowledge of SRs on the postintervention questionnaire after the participants had read the educational materials. After the training, the educational intervention group had 23% more correct answers than the PRISMA checklist<br>group (ie, the size of the effect expressed in RR was 1.23).<br><br>An RCT by Sánchez-Mendiola et al [15] showed a significant effect of EBM education on the final knowledge of EBM among medical students, with a 25.9% increase in correct answers in the knowledge test about EBM [15]."		
**22-ii) Highlight unanswered new questions, suggest future research**		
Other information		
**23) CONSORT: Registration number and name of trial registry**		
OSF Registry, available at: https://osf.io/rymvc		
**24) CONSORT: Where the full trial protocol can be accessed, if available**		
Open Science Framework; https://osf.io/rymvc		
**25) CONSORT: Sources of funding and other support (such as supply of drugs), role of funders**		
Open Science Framework; https://osf.io/x2mf5/		
**X26-i) Comment on ethics committee approval**		
**x26-ii) Outline informed consent procedures**		
**X26-iii) Safety and security procedures**		
**X27-i) State the relation of the study team towards the system being evaluated**		
