# Supplementary material for: Web-Based Educational Intervention to Improve Knowledge of Systematic Reviews Among Health Science Professionals: Randomized Controlled Trial
Source: J Med Internet Res. 2022 Aug 25;24(8):e37000. doi: 10.2196/37000 (PMC9459937; doi:10.2196/37000)
Supplement: Multimedia Appendix 2 [file jmir_v24i8e37000_app2.docx]

**Supplementary file 2. Text of the first e-mail and reminders to invite participants to a randomized controlled trial**

Message subject: Education on systematic reviews of the literature: an invitation to participate in the study

Dear colleagues,

We invite you to participate in a brief online education about systematic reviews of the literature. You can access online education from any location at any time of the day.

By participating in this education, you are also participating in a study about the usefulness of two types of such education. The study is designed as a randomized controlled trial. Participants who agree to participate in the study will be allocated into two groups. As a participant, you will not be able to know in advance the group in which you will be allocated. Both groups will receive an initial questionnaire, then the groups will receive different educational materials, and after reading the material, all participants will receive the final questionnaires. The education will be conducted online, and the maximum expected duration of your participation is 30 minutes. If you complete the education, you can (if you wish) apply for a certificate of attendance at the end of the online interface. Cochrane Croatia will issue the certificate. If you agree to participate, you are giving your informed consent to participate in the education and the study by entering the questionnaire form.

To access the questionnaire, please click on the link below:

Link XYXYXYXY

Please answer all questions spontaneously and honestly.

The study was approved by the Ethics Committees of all participating institutions. There are no risks associated with this study, i.e. the level of discomfort in this research is not higher than the one you experience in everyday life.

Participation in the study is anonymous. Even if you decide to leave your name and e-mail address to get the certificate, anonymized data set will be used for data analysis. The collected data will be used only for this study and will be stored in digital form on the computer of the research administrator, protected by a password.

Only researchers will have access to the data. The results will be used and published to improve the quality of teaching, write scientific articles and lectures.

Participation in the study is voluntary. You have the right to withdraw from the study at any time without any consequences.

Under applicable law, you have the right to access your personal data, correct, delete, restrict the processing and portability of personal data before the publication of results, and the right to object to the processing and lodge a complaint with the Croatian Personal Data Protection Agency.

If you want to be informed about the results and conclusions of the study or have questions or requests regarding the study, please contact the principal investigator (Prof. Livia Puljak, MD, PhD; livia.puljak@unicath.hr).

If you have any complaints about the study procedures or are concerned about something you experienced during the study, please contact the principal investigator (Prof. Livia Puljak, MD, PhD; livia.puljak@unicath.hr).

We would appreciate if you could accept our invitation and participate in the study.

Sincerely,

Marina Krnić Martinić, M.D.
