# Supplementary material for: Web-Based Educational Intervention to Improve Knowledge of Systematic Reviews Among Health Science Professionals: Randomized Controlled Trial
Source: J Med Internet Res. 2022 Aug 25;24(8):e37000. doi: 10.2196/37000 (PMC9459937; doi:10.2196/37000)
Supplement: Multimedia Appendix 3 [file jmir_v24i8e37000_app3.docx]

**Supplementary file 3: Educational intervention**

Please, now read the 11 short modules with information on systematic reviews:

**1. Definition of evidence-based medicine**

Evidence-based medicine is an approach to health care that combines the experience of health professionals with the wishes and needs of patients and the best scientific evidence.

**2. Hierarchy of evidence in medicine**

Not all evidence in medicine is considered equally valuable. The hierarchy of evidence in medicine is depicted as a pyramid; at the top of the pyramid, there is evidence we can trust more (evidence which is more reliable and with a lower risk of bias), and at the bottom is evidence that we can trust less (less reliable, with a higher risk of bias).

At the top of this pyramid are randomized clinical trials conducted on humans and systematic reviews of the literature that combine the results of individual studies. In the middle of the pyramid are perceptual research, and at the bottom of the pyramid are personal opinions, research on animals, cells, and models.

**3. Systematic review of the literature**

A systematic review of the literature is a type of research that combines the results of research conducted on a specific, predefined topic. Such a review of the literature is called “systematic” because there is a demanding methodology that needs to be followed for a systematic review to be properly conducted. Such research includes asking a clinical question, systematically searching the literature, analyzing data, and drawing conclusions based on all research available on a specific topic. All these criteria are agreed upon and defined in advance, before the start of the research.

While conducting a systematic review, multiple scientific research databases should be searched to increase the likelihood of finding all studies available on a defined topic.

A series of steps during the conduct of a systematic review should be done by two authors independently, after which the results of their work are compared, in order to avoid accidental errors and bias.

The steps in conducting a systematic review are:

1. Define the question
2. Plan eligibility criteria
3. Plan methods
4. Search for studies
5. Apply eligibility criteria
6. Collect data
7. Assess studies for risk of bias
8. Analyze and present results
9. Interpret results and draw conclusions
10. Improve and update review

The six key features of a systematic review are:

1. A research question is defined
2. Listed literature sources searched, with repeatable search strategy (naming of databases, naming of search platforms, search dates and complete search strategy)
3. Listed criteria for inclusion and exclusion of research
4. Listed selection (screening) methods
5. Critically evaluates and reports on the quality/risk of bias of the studies included
6. Provides information on the analysis and synthesis of data that allow repeatability of results.

**4. Beginning of a systematic review production: asking a clinical question**

A systematic review of the literature begins with asking the research question, which will be investigated. The research question usually contains the so-called PICO elements (Patient/health problem, Intervention, Comparison/control, Outcome), i.e. a description of the patients’ characteristics, the intervention to be investigated, the control with which the intervention will be compared (comparator) and the planned outcomes. An example of a research question for a systematic review of the literature is:

Can a single dose of ibuprofen 400 mg relieve postoperative pain in adults after wisdom tooth extraction, compared to placebo?

In this research question, patients are adults who need a wisdom tooth extraction, the study intervention is 400 mg of ibuprofen in a single dose, the comparison is placebo, and the planned outcome is the alleviation of postoperative pain.

**5. Writing a systematic review protocol and registering the protocol**

When defining a research question, it is necessary first to write a systematic review protocol, which will describe in detail the planned methods, including:

- indication of inclusion and exclusion criteria (types of research to be included, characteristics of prticipants, types of comparisons, types of outcomes)

- naming the databases to be searched and by which search strategy,

- research selection methods that meet the inclusion criteria,

- the way in which the data from the included surveys will be extracted,

- a description of the methods of critical assessment of quality, i.e. the risk of bias in the included research

- method of assessing the credibility of evidence,

- information on data analysis and synthesis (statistical analysis, meta-analysis)

- method of handling missing data,

- method of assessing the heterogeneity (diversity) of the included research,

- analysis of publishing bias,

- sensitivity analysis.

It is desirable to publish or register the protocol in a publicly available register on the Internet so that the planned methods of a systematic review are publicly available and transparent.

**6. Literature search**

In a systematic review of the literature, it is necessary to search at least two electronic databases of scientific research, e.g. MEDLINE or PubMed, and Embase. Searching a larger number of scientific databases increases the probability of finding a larger number of papers on a given topic. Some databases of scientific papers are specialized and are used only for certain topics. The search of electronic databases should be supplemented by searching additional sources of literature, such as registers of clinical trials, dissertations, unpublished research, conference abstracts, and references and citations of included research.

Complex search strategies are used to search the literature, including the use of predefined medical terms and free words in the text. An information specialist should be involved in developing a complex search strategy.

**7. Screening the literature**

After searching the literature, at least two authors should first independently review all titles and abstracts obtained by searching and exclude those that certainly do not meet the inclusion criteria. The full texts of the records retained in the first phase as eligible or potentially eligible are analyzed in the next phase. After full-text analysis, only studies that meet the eligibility criteria are included. The duration of literature screening obtained by searching can be very long, depending on the complexity of the topic. Literature search can yield thousands or tens of thousands of records that need to be screened.

**9. Research quality and risk of bias**

The quality of all studies included in the systematic review needs to be assessed to determine the potential risk of bias. Namely, not all research is done equally well, and poor research is not reliable. Therefore, to adequately assess the reliability of the data collected by a systematic review, it is necessary to know whether we can trust the included studies. The risk of bias assessment includes an analysis of multiple methodological features of the included studies, from randomization into groups, concealment of randomization, concealment of the obtained intervention and measurement of outcomes, participants’ attrition, selective reporting and any other source of bias. The risk of bias assessment tells us whether the methods were inappropriate or unclear and whether they could have affected the reliability of the data. Standardized instruments are used for this assessment.

**10. Data analysis**

If the studies included in a systematic review are sufficiently similar, a statistical analysis of the data called meta-analysis can be used. In a meta-analysis, the results collected from several studies are analyzed together. A meta-analysis, therefore, allows us to numerically aggregate the results of a larger number of studies and thus to obtain more reliable evidence than any of these studies separately. The results of the meta-analysis are presented in a graph called a forest plot.

The forest plot is a graphical representation of a meta-analysis. It shows results from included studies for one outcome. Each horizontal line represents a single study included in the systematic review; the length of the line represents the 95% confidence interval, and the square on the line is the main result of a study for the analyzed outcome. The figure you can see hereby shows a meta-analysis that included five studies (five horizontal blue lines with squares). At the bottom of these lines is the summary result of a meta-analysis, based on the results of all included studies, depicted as a black rhombus (we call it a "diamond"), which shows the cumulative effect of those five studies.

The vertical line in the forest plot represents the "line of no effect". If the results of individual studies or the diamond are on one side of that line, it means that the outcome is better in one group (in this case, it is exercise), and if the results are on the opposite side of the line, it means that the outcome was better in the other group (in this case it was the control group). If the line or diamond touches the "line of no effect ", it means that there is no significant difference between the intervention and control group.

Although the lines of some studies in the example shown in this figure cross the “line of no effect”, when we do a meta-analysis, we see that the diamond is on the left side of the line. Thus, the combined results of all five studies show that the outcome is better in the group that received the intervention (exercise).


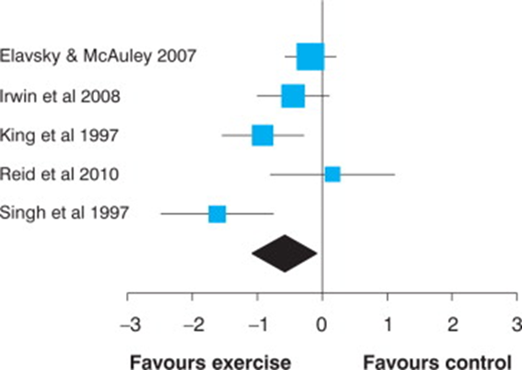


Data from meta-analyses can be used to assess the risk of publication bias, which is presented by a graph called a funnel plot. Namely, the funnel plot is a scatterplot of treatment effect against a measure of study precision. In the diagram, each dot represents one study. The effect estimates from smaller studies should be less accurate and spread wider at the bottom of the diagram, while the effect estimates from larger studies should be more accurate and should narrow towards the top of the diagram. Ideally, the dots should be arranged symmetrically in the shape of a funnel or pyramid. The asymmetric arrangement of dots on the funnel plot indicates the possibility of bias in the publication of the results.


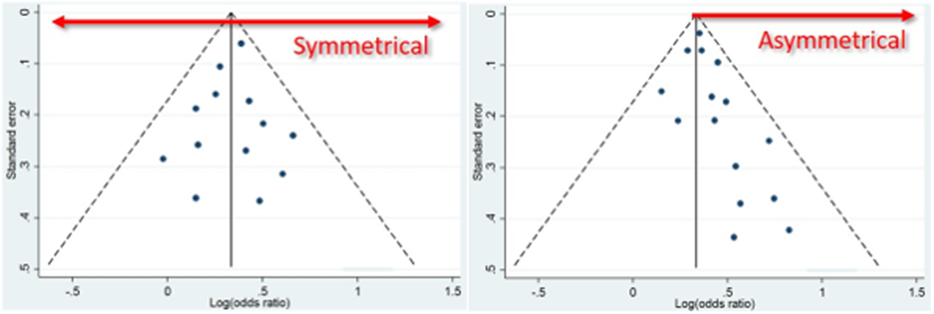


**11. Interpretation of data - conclusion for clinical practice and recommendations for future research**

After analyzing the data, the data is interpreted, and conclusions are drawn for future clinical practice and recommendations made for future research. While making conclusions, the risk of bias assessment and certainty of the evidence for specific outcomes should be taken into consideration. If the included studies are not of high quality and reliable, then it is important to conclude that we cannot fully trust them and that new research on this topic is necessary.

A systematic review clearly and accurately displays all available studies on a particular topic, from multiple databases and with reduced researcher bias; puts the results in context by comparing different researches, and helps to determine which new researches are needed by outlining what has been done, how well and with what results.
