# Supplementary material for: Web-Based Educational Intervention to Improve Knowledge of Systematic Reviews Among Health Science Professionals: Randomized Controlled Trial
Source: J Med Internet Res. 2022 Aug 25;24(8):e37000. doi: 10.2196/37000 (PMC9459937; doi:10.2196/37000)
Supplement: Multimedia Appendix 5 [file jmir_v24i8e37000_app5.docx]

**Supplementary file 5: The text of the pre-intervention and post-intervention questionnaires**

***First page of SurveyMonkey platform: an introductory statement***

Dear Colleagues,

Thank you for participating in the study. By following the link, you are entering the interface for conducting the education, confirming that you are participating in the study voluntarily and that you are giving informed consent for participation.

**Pre-intervention questionnaire**

1. Select your University Department of Health Studies:

1. Catholic University of Croatia

2. University Department of Health Studies Split

3. University Department of Health Studies Zadar

4. University of Dubrovnik, Nursing Studies

5. University North

6. Faculty of Dental Medicine and Health, University of Osijek

7. Faculty of Health Studies, University of Rijeka

8. Medical School, University of Zagreb

2. Your field of studies:

Nursing

Physiotherapy

Radiological technologies

Medical laboratory diagnostics

Midwifery

3. Your study year:

1

2

3

4. Are you currently employed?

Yes

No

5. Are you currently employed as a healthcare worker?

Yes

No

5a. If yes, how many years of experience working as a healthcare worker do you have? _______ (years)

6. How old are you: ___________years

7. Your sex:

Male

Female

I do not wish to declare

8. How would you rate your knowledge about evidence-based medicine and scientific methodology? Use grades from 1 (insufficient knowledge) to 5 (excellent knowledge).

1 2 3 4 5

9. Have you ever heard of a type of study/scientific article called systematic review?

Yes

No

If your answer to question #9 was yes:

10. Where have you heard about systematic reviews: ______________ (enter an answer)

11. Have you ever read a systematic review?

Yes

No

Other answer: _______________

12. Have you ever participated in producing a systematic review?

Yes

No

Please assess the following statements:

13. In a systematic review, it is enough to search one database

Yes

No

I am not certain

I do not know

14. A true systematic review can be done by a single author

Yes

No

I am not certain

I do not know

15. Systematic reviews must contain a meta-analysis

Yes

No

I am not certain

I do not know

16. While producing a systematic review, two authors should independently assess studies found in the literature search and should independently extract data from the included studies.

Yes

No

I am not certain

I do not know

17. A systematic review should provide a list of included and excluded studies.

Yes

No

I am not certain

I do not know

18. A systematic review should contain a quality assessment of the included studies.

Yes

No

I am not certain

I do not know

19. If a statistical analysis called meta-analysis is performed, an assessment of heterogeneity (diversity) should be performed to verify if the studies are comparable.

Yes

No

I am not certain

I do not know

20. Meta-analysis results are displayed as a graph called funnel plot:

Yes

No

I am not certain

I do not know

21. Publication bias assessment results are displayed as a graph called forest plot.

Yes

No

I am not certain

I do not know

22. Read the following characteristics and state if you agree whether a systematic review should contain those characteristics. Express your agreement with a number on a scale from 1 to 5 that best suits your opinion, where 1 means "I do not agree at all" and 5 "I completely agree".

i) A research question has been defined 1 2 3 4 5

ii) Listed sources of literature searched, with repeatable search strategy (naming of databases, naming of search platforms, search date and complete search strategy) 1 2 3 4 5

iii) Listed criteria for inclusion and exclusion of research 1 2 3 4 5

iv) Listed selection methods 1 2 3 4 5

v) Critically evaluates and reports on the quality/risk of bias of the included studies 1 2 3 4 5

vi) Provides information on data analysis and synthesis that allows repeatability of results 1 2 3 4 5

**Post -intervention questionnaire**

After reading the information shown, please answer the following questions:

Please assess the following statements:

13. In a systematic review, it is enough to search one database

Yes

No

I am not certain

I do not know

14. A true systematic review can be done by a single author

Yes

No

I am not certain

I do not know

15. Systematic reviews must contain a meta-analysis

Yes

No

I am not certain

I do not know

16. While producing a systematic review, two authors should independently assess studies found in the literature search and should independently extract data from the included studies.

Yes

No

I am not certain

I do not know

17. A systematic review should provide a list of included and excluded studies.

Yes

No

I am not certain

I do not know

18. A systematic review should contain a quality assessment of the included studies.

Yes

No

I am not certain

I do not know

19. If a statistical analysis called meta-analysis is performed, an assessment of heterogeneity (diversity) should be performed to verify if the studies are comparable.

Yes

No

I am not certain

I do not know

20. Meta-analysis results are displayed as a graph called funnel plot:

Yes

No

I am not certain

I do not know

21. Publication bias assessment results are displayed as a graph called forest plot.

Yes

No

I am not certain

I do not know

22. Read the following characteristics and state if you agree whether a systematic review should contain those characteristics. Express your agreement with a number on a scale from 1 to 5 that best suits your opinion, where 1 means "I do not agree at all" and 5 "I completely agree".

i) A research question has been defined 1 2 3 4 5

ii) Listed sources of literature searched, with repeatable search strategy (naming of databases, naming of search platforms, search date and complete search strategy) 1 2 3 4 5

iii) Listed criteria for inclusion and exclusion of research 1 2 3 4 5

iv) Listed selection methods 1 2 3 4 5

v) Critically evaluates and reports on the quality/risk of bias of the included studies 1 2 3 4 5

vi) Provides information on data analysis and synthesis that allows repeatability of results 1 2 3 4 5

11. How would you use systematic reviews in your clinical practice? ______________ (please state)

12. If you had a need to look for information to solve a clinical problem, where would you look for that information? (multiple answers are possible)

1. From colleagues at work
2. In books
3. In scientific literature
4. In a systematic review
5. On an Internet search engine such as Google
6. Somewhere else: _________________________ (please state)

Now please evaluate the four abstracts from the literature. For each abstract, evaluate whether it describes a systematic review of the literature or not.

1. Summary 1. Is this a summary of a systematic review of the literature? Yes / No
2. Summary 2. Is this a summary of a systematic review of the literature? Yes / No
3. Summary 3. Is this a summary of a systematic review of the literature? Yes / No
4. Summary 4. Is this a summary of a systematic review of the literature? Yes / No

**If you want to get a certificate for attending this training,** please leave your name and e-mail address. We will not use your name and e-mail address for any purpose other than sending the certificate; after completing the study, we will delete your name and e-mail address from the data collected.

Name and surname:

E-mail address:

**Thank you for participating in the education and research**
