# Supplementary material for: Web-Based Educational Intervention to Improve Knowledge of Systematic Reviews Among Health Science Professionals: Randomized Controlled Trial
Source: J Med Internet Res. 2022 Aug 25;24(8):e37000. doi: 10.2196/37000 (PMC9459937; doi:10.2196/37000)
Supplement: Multimedia Appendix 6 [file jmir_v24i8e37000_app6.docx]

**Supplementary file 6: Four selected article abstracts for assessment**

**Abstract 1**

**Surgical interventions for chronic rhinosinusitis with nasal polyps [25]**

**Abstract**

**Background:**Surgical treatment of chronic rhinosinusitis with nasal polyps is an established treatment for medically resistant nasal polyp disease. Whether a nasal polypectomy with additional sinus dissection offers any advantage over an isolated nasal polypectomy has not been systematically reviewed.

**Objectives:**To assess the effectiveness of simple polyp surgery versus more extensive surgical clearance in chronic rhinosinusitis with nasal polyps.

**Search methods:**We searched the Cochrane Ear, Nose and Throat Disorders Group Trials Register; the Cochrane Central Register of Controlled Trials (CENTRAL 2014, Issue 1); PubMed; EMBASE; CINAHL; Web of Science; Cambridge Scientific Abstracts; ICTRP and additional sources for published and unpublished trials. The date of the search was 20 February 2014.

**Selection criteria:**Randomized and quasi-randomized controlled trials in patients over 16 with chronic rhinosinusitis with nasal polyps, who have failed a course of medical management and who have not previously undergone any previous surgical intervention for their nasal disease. Studies compared nasal polypectomy with more extensive sinus clearance in this patient cohort.

**Data collection and analysis:**We used the standard methodological procedures expected by The Cochrane Collaboration.

**Main results:**We identified no trials which met our inclusion criteria. Six controlled trials (five randomized) met some but not all of the inclusion criteria and were therefore excluded from the review.

**Authors' conclusions:**We are unable to reach any conclusions as to whether isolated nasal polypectomy or more extensive sinus surgery is a superior surgical treatment modality for chronic rhinosinusitis with nasal polyps. There is a need for high-quality randomized controlled trials to assess whether additional sinus surgery confers any benefit when compared to nasal polypectomy performed in isolation.

**Abstract 2**

**Garlic for the common cold [26]**

**Abstract**

**Background:**Garlic is alleged to have antimicrobial and antiviral properties that relieve the common cold, among other beneficial effects. There is widespread usage of garlic supplements. The common cold is associated with significant morbidity and economic consequences. On average, children have six to eight colds per year and adults have two to four.

**Objectives:**To determine whether garlic (allium sativum) is effective for either the prevention or treatment of the common cold, when compared to placebo, no treatment or other treatments.

**Search methods:**We searched the Cochrane Central Register of Controlled Trials (CENTRAL) (2011, Issue 4), which includes the Cochrane Acute Respiratory Infections Group Specialized Register, OLDMEDLINE (1950 to 1965), MEDLINE (January 1966 to November week 3, 2011), EMBASE (1974 to December 2011) and AMED (1985 to December 2011).

**Selection criteria:**Randomized controlled trials of common cold prevention and treatment comparing garlic with placebo, no treatment or standard treatment.

**Data collection and analysis:**Two review authors independently reviewed and selected trials from searches, assessed and rated study quality and extracted relevant data.

**Main results:**Of the six trials identified as potentially relevant from our searches, only one trial met the inclusion criteria. This trial randomly assigned 146 participants to either a garlic supplement (with 180 mg of allicin content) or a placebo (once daily) for 12 weeks. The trial reported 24 occurrences of the common cold in the garlic intervention group compared with 65 in the placebo group (P < 0.001), resulting in fewer days of illness in the garlic group compared with the placebo group (111 versus 366). The number of days to recovery from an occurrence of the common cold was similar in both groups (4.63 versus 5.63). Only one trial met the inclusion criteria, therefore limited conclusions can be drawn. The trial relied on self-reported episodes of the common cold but was of reasonable quality in terms of randomization and allocation concealment. Adverse effects included rash and odor.

**Authors' conclusions:**There is insufficient clinical trial evidence regarding the effects of garlic in preventing or treating the common cold. A single trial suggested that garlic may prevent occurrences of the common cold, but more studies are needed to validate this finding. Claims of effectiveness appear to rely largely on poor-quality evidence.

**Abstract 3**

**Lumbar medial branch neurotomy for the treatment of back pain [27]**

**Abstract**

**Background:**Confusion persists concerning the nature and efficacy of procedures variously known as facet denervation, lumbar medial branch radiofrequency neurotomy, and radiofrequency neurotomy or denervation for the treatment of back pain. Systematic reviews have not recognized the importance of patient selection and correct surgical technique when appraising the literature. As a result, negative conclusions about procedures have been drawn because lack of efficacy of one procedure has been misattributed to other, cognate, but different procedures.

**Objectives:**To demonstrate how the rationale and efficacy of lumbar medial branch neurotomy depends critically on correct selection of patients and use of surgically correct technique.

**Methods:**A review and description of the available evidence, drawn from the personal libraries of the authors and from the bibliographies of systematic reviews.

**Results:**Three studies, commonly accepted as evidence of lack of effectiveness, were not valid tests of lumbar medial branch neurotomy because of errors in selection of patients or errors in surgical technique, or both. Two descriptive studies and three controlled studies that used valid or acceptable techniques consistently showed that lumbar medial branch neurotomy had positive effects on pain and disability. All valid, randomized controlled trials showed medial branch neurotomy to be more effective than sham treatment.

**Discussion:**Negative results have been reported only in studies that selected inappropriate patients or used surgically inaccurate techniques. All valid studies showed positive outcomes that cannot be attributed to placebo. Inappropriate conclusions have been drawn by systematic reviews that misrepresent invalid studies as providing evidence against the efficacy of lumbar medial branch neurotomy.

**Abstract 4**

**Nurses' preparedness for infectious disease outbreaks: A literature review and synthesis of qualitative evidence [28]**

**Abstract**

**Aims and objectives:**To explore the core components that constitute nurses' preparedness in an epidemic event.

**Background:**Healthcare service providers have worked to augment efforts to protect the public from the impact of epidemic events. While constituting the major healthcare taskforce, nurses are frequently tasked with fronting the response to an infectious disease outbreak. Given the crucial role of nurses in the management of prevailing epidemics, the constituents of their preparedness in epidemic events should be identified.

**Design:**A systematic search was undertaken to identify eligible studies from the literature. A narrative synthesis approach was employed to extract and synthesize findings of the reviewed qualitative studies.

**Methods:**Seven qualitative studies on nurses' experience and perceptions of epidemic events were examined for scientific quality using the Critical Appraisal Skills Program Qualitative Checklist. Findings of these studies were synthesized adopting a narrative synthesis approach.

**Results:** Three interplaying themes were identified as follows: (i) personal resources, (ii) workplace resources and (iii) situational influences. The findings suggest that an effective epidemic outbreak response would require further effort to reinforce the interplay between individual nurses, healthcare institutions and the governments.

**Conclusions:** The practical interplay among individual nurses, healthcare institutions and the governments is crucial in establishing an effective epidemic response. Further research on the understanding of the dynamic process of preparedness development is recommended to set future directions in research.

**Relevance to clinical practice:** This study offers important insights for devising future strategies in enhancing nurses' preparedness and response to an epidemic event. These include recommendations on providing education and training to nurses regarding infectious diseases, fostering institutional assistance and support in an outbreak and revising government policies and planning.

**Keywords:** communicable diseases; disease outbreaks; nurses; preparedness.
